# Recent advances in gut microbiota and thyroid disease: pathogenesis and therapeutics in autoimmune, neoplastic, and nodular conditions

**DOI:** 10.3389/fcimb.2024.1465928

**Published:** 2024-12-24

**Authors:** Lihua Fang, Jie Ning

**Affiliations:** Department of Endocrinology, Shenzhen Longhua District Central Hospital, Shenzhen, Guangdong, China

**Keywords:** gut microbiota, thyroid disease, SCFAs, cytokine, probiotics therapy

## Abstract

This review synthesizes key findings from the past five years of experimental literature, elucidating the gut microbiome’s significant influence on the pathogenesis of thyroid diseases. A pronounced shift in the gut microbiota composition has been consistently observed, with a significant reduction in bacteria such as *Bifidobacterium*, *Bacillaceae*, *Megamonas*, and *Clostridium*, and a notable increase in bacteria, including *Bacteroides*, *Proteobacteria*, *Actinobacteria*, *Desulfobacterota*, and *Klebsiella*. These alterations are implicated in the development and progression of thyroid diseases by impacting metabolic pathways including bile acid and cytokine production, including a decrease in short-chain fatty acids (SCFAs) that are crucial for immune regulation and thyroid hormone homeostasis. The review also highlights the therapeutic implications of probiotics in managing thyroid conditions. Evidence suggests that probiotic adjunct therapy can modulate the gut microbiota, leading to improvements in thyroid function and patient outcomes. The use of specific probiotic strains, such as *Lactiplantibacillus plantarum* 299v and *Bifidobacterium longum*, has demonstrated potential in enhancing the effects of traditional treatments and possibly restoring a balanced gut microbiota. Notably, fecal microbiota transplantation (FMT) has emerged as a promising intervention in Graves’ Disease (GD), demonstrating the potential to recalibrate the gut microbiota, thereby influencing neurotransmitters and trace elements via the gut-brain and gut-thyroid axes. The integration of microbiome-based therapies with traditional treatments is anticipated to usher in a new era of personalized thyroid disease management, offering a more nuanced approach to patient care. By integrating this body of work, the review offers an innovative perspective on the gut microbiome’s broad impact on thyroid diseases and the therapeutic applications of probiotics.

## 1 Introduction

**Figure 1:**
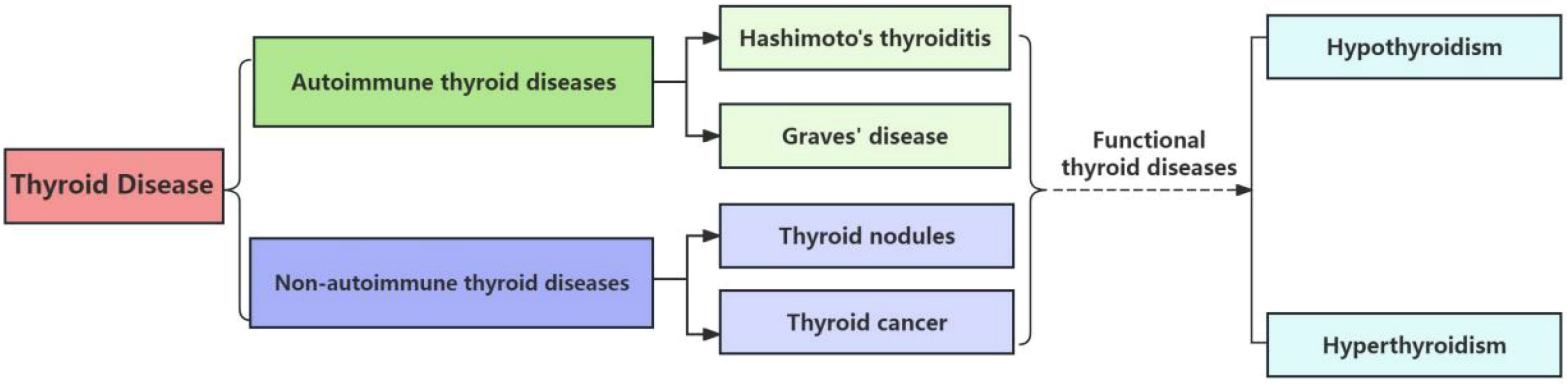
Scheme of thyroid diseases classification. This scheme categorizes the primary types of thyroid diseases, including autoimmune, neoplastic, and nodular conditions. The dashed lines indicate the progression from Hashimoto’s Thyroiditis, an autoimmune condition, to hypothyroidism, and from the other three diseases under review, which encompass both autoimmune and non-autoimmune etiologies, to hyperthyroidism.

Thyroid diseases, a spectrum of conditions resulting from pathological alterations in the thyroid gland, are increasingly prevalent globally (Elmisbah et al., 2024). They encompass a range of disorders, including autoimmune and non-autoimmune thyroid diseases as depicted in **Figure 1**, each with distinct pathophysiological mechanisms and clinical implications (Vargas-Uricoechea, 2024). Hypothyroidism and hyperthyroidism, in particular, stand out as the most commonly encountered conditions, affecting millions of individuals worldwide. These diseases not only impact metabolic regulation but also hold significant implications for overall health and well-being (Xu et al., 2024).

As our understanding of the gut microbiota deepens, numerous studies have indicated that the microorganisms living within the human body play a significant role in different thyroid diseases such as hypothyroidism and autoimmune thyroid diseases (Stramazzo et al., 2023).Thyroid dysfunction (e.g. Graves’ disease and Hashimoto’s thyroiditis) (Kyriacou et al., 2015) can manifest with a variety of gastrointestinal symptoms, including dysphagia, heartburn (Gianoukakis et al., 2021), indigestion, reduced acid production, nausea or vomiting, gallbladder discomfort, abdominal discomfort, bloating, flatulence, diarrhea, constipation, and general digestive discomfort (Xu et al., 2024), including irritable bowel syndrome (IBS) (Borrego-Ruiz and Borrego, 2024). One of the most common sources of inflammation leading to autoimmune diseases is increased intestinal permeability or “leaky gut” (Cayres et al., 2021; Zheng et al., 2021).

The human lower intestine is home to an enormous population of bacteria, numbering between 10 (Xiao et al., 2020) and 10 (Rivière et al., 2016), which significantly exceeds the count of human eukaryotic cells (Gill et al., 2006), estimated to be around 10 (Eckburg et al., 2005). Comprehensive analyses of the gut microbiota have unveiled a rich tapestry of approximately 1000 distinct bacterial species (Qin et al., 2010). Taxonomically, the phyla Firmicutes and Bacteroides predominate, constituting 70% to 75% of the gut microbiome, with additional contributions from Proteobacteria, Actinobacteria, Fusobacteria, and Verrucomicrobia (Eckburg et al., 2005). Beneficial bacteria from the phyla Bifidobacterium and Lactobacillus produce short-chain fatty acids (SCFA) that are crucial for immune regulation and maintaining thyroid hormone balance, while also preserving the intestinal barrier integrity (Xiao et al., 2020). In contrast, harmful bacteria like Bacteroides, Proteobacteria, and Actinobacteria disrupt SCFA balance, decrease their production, and promote inflammation, impacting thyroid function and health (Rivière et al., 2016). These harmful bacteria can increase gut permeability, allowing translocation of components like lipopolysaccharides (LPS) (Ghosh et al., 2020), which activate immune cells and trigger inflammatory responses. This could potentially lead to autoimmune thyroid diseases characterized by the production of autoantibodies against thyroid peroxidase (TPO) and thyroglobulin (Tg) (Iwamoto et al., 2023).

Intestinal mucosa acts as a “customs” checkpoint. The intestinal mucosa (inner lining of the gut) allows nutrients from food to pass into the submucosal layer, facilitating our absorption of these nutrients, while excluding potential harmful substances and bacterial fragments from the food that could trigger inflammation and immune responses in the submucosal layer (Toskas et al., 2024). The thyroid gland requires key nutrients (Shulhai et al., 2024) such as iodine for hormone synthesis, selenium for enzyme function, and zinc and iron for metabolic processes, with deficiencies or imbalances potentially leading to thyroid dysfunction (Kowalczyk et al., 2017). The gut microbiota affects the absorption of nutrients, including trace elements (Abulizi et al., 2019). The microbiota can process and break down food for better nutrient absorption (Avila-Arias et al., 2023). At the same time, the gut microbiota itself requires nutrients to sustain life (Nannini and Amedei, 2024). Some bacteria feed on sugars and carbohydrates, while others feed on fats and other minerals (Colella et al., 2023). When these microbes are imbalanced, it can affect nutrient acquisition. Patients with autoimmune thyroid diseases often lack micronutrients, among which selenium, iodine, and zinc are particularly important for the thyroid (El-Zawawy et al., 2021). If there is inflammation or dysbiosis in the body, the absorption of these nutrients may be affected. The production of primary bile acids in the gallbladder is also important (Long et al., 2023). These are secreted from the gallbladder into the small intestine after the consumption of fats, where gut bacteria metabolize them into so-called “secondary bile acids,” thereby increasing the activity of deiodinase enzymes (Rowe and Winston, 2024). The study by Watanabe et al (Watanabe et al., 2006) further illustrates this mechanism, showing that bile acids increase energy expenditure in brown adipose tissue by promoting intracellular thyroid hormone activation, which is critically dependent on the induction of the thyroid hormone activating enzyme type 2 iodothyronine deiodinase (D2). This indicates that bile acids, through their interaction with deiodinases, play a role in thyroid hormone metabolism and overall thyroid health. More importantly, these bile acids depend on the aforementioned mineral selenium, and patients with intestinal and thyroid health problems are often depleted of selenium (Amini et al., 2023). Hypothyroidism can also hinder the gallbladder’s bile flow, further affecting the T4/T3 conversion (Jo et al., 2022). This bile also has natural antibacterial properties, which may further explain the link between thyroid diseases and specific bacterial gut infections, such as SIBO (Docimo et al., 2020).

Meanwhile, the gut serves another vital function as the host for 70% of the body’s immune tissue (Vakaloloma et al., 2023). This part of the immune system is collectively referred to as gut-associated lymphoid tissue (GALT) (Bemark et al., 2024). GALT includes various types of lymphoid tissues that store immune cells, such as T and B lymphocytes, which attack and produce antibodies against antigens—molecules that the immune system recognizes as potential threats (Montorsi et al., 2024). A healthy gut microbiota can influence the immune system and has a significant impact on thyroid function, especially in autoimmune thyroid diseases such as Hashimoto’s thyroiditis (HT) and Graves’ disease (Sprouse et al., 2019). Individuals with autoimmune thyroid diseases are 4-5 times more likely to have celiac disease than the general population (Stazi and Trinti, 2008). This can be explained by impaired gut barrier function, which allows antigens to pass through more easily and trigger autoimmune diseases in genetically susceptible individuals.

Therefore, the interplay between the thyroid gland and the gut has emerged as a pivotal area of interest in endocrinology (Borrego-Ruiz and Borrego, 2024). The gut-thyroid axis is a burgeoning field of study, with research uncovering the intricate relationship between the microbiota and the endocrine system. In this review, we delve into the latest advancements in the understanding of the gut microbiome’s role in thyroid diseases, focusing on the most recent five-year span of experimental studies. We majorly provide an overview of how the gut microbiome influences autoimmune thyroid diseases such as Hashimoto’s and Graves’, as well as its broader impact on non-autoimmune conditions like thyroid nodules and thyroid cancer. In this review, we will focus on these last four diseases, given their potential to result in functional thyroid disorders, including hypothyroidism and hyperthyroidism as indicated in **Figure 1**, we focus our discussion on these four diseases in this article. Moreover, we explore the therapeutic potential of probiotic therapy and fecal microbiota transplantation, a cutting-edge intervention that holds promise for restoring thyroid health. By examining the current body of research, we aim to highlight the innovative approaches and significance of microbiome-based therapies in the management of thyroid diseases, emphasizing the progress and potential of this emerging field.

## 2 Materials and methods

For our research methodology, we meticulously searched the NCBI - PubMed database for articles related to “gut microbiota and each thyroid diseases, “specifically targeting experimental studies published within the past five years and omitting review articles. We then categorized and synthesized the data to understand the relationship between the gut microbiota and four distinct thyroid conditions. Following this, we conducted an analogous search with the query “probiotic and various thyroid diseases,” filtering for recent experimental research over the last five years and excluding review articles. This dual approach not only highlights our analytical prowess but also ensures the novelty and innovation of our comprehensive analysis.

## 3 Results

### 3.1 The microbiota’s broader impact on autoimmune thyroid diseases

Autoimmune thyroid diseases, such as Hashimoto’s thyroiditis and Graves’ disease, represent some of the most prevalent organ-specific autoimmune conditions globally, affecting over 5% of the population (Stramazzo et al., 2023). These conditions are characterized by an immune response against the thyroid gland, leading to inflammation and dysfunction. The gastrointestinal system, acting as a significant endocrine entity, is instrumental in the pathogenesis of autoimmune thyroid disorders. The phenomenon of molecular mimicry, where there is a resemblance between certain microbial and self-antigens, can potentially trigger autoimmune reactions in susceptible individuals under specific conditions (Benvenga and Guarneri, 2016).

#### 3.1.1 Gut microbiome and Hashimoto’s thyroiditis

Hashimoto’s thyroiditis, also known as chronic lymphocytic thyroiditis, is primarily associated with the presence of thyroid peroxidase antibodies and, to a lesser extent, thyroglobulin antibodies. These antibodies are indicative of an autoimmune attack on the thyroid gland, leading to inflammation and impaired thyroid hormone production, typically resulting in hypothyroidism (Virili et al., 2018). The most typical symptoms of Hashimoto’s Thyroiditis include local compression effects such as dysphonia, dyspnea, and dysphagia, along with systemic manifestations of hypothyroidism like fatigue, weight gain, and cold intolerance (Ralli et al., 2020). Its etiology is multifactorial, involving genetic predisposition, environmental factors, and an interplay between the immune system and the thyroid gland (Klubo-Gwiezdzinska and Wartofsky, 2022). Recent studies suggest that the incidence and prevalence of HT are on the rise, with a growing body of evidence implicating factors such as iodine intake, selenium deficiency, and exposure to environmental pollutants (Weetman, 2021). The disease is more common in women and tends to increase with age, with the highest incidence rates observed in middle-aged and older adults (Konturek et al., 2016).

Recent research has shed light on the intricate connection between HT and the gut microbiome, with a particular focus on the changes in the gut’s bacterial composition. A comprehensive statistical analysis of the latest five-year research papers has been performed, and the results are neatly summarized in **Table 1**. The findings consistently indicate a significant shift in the gut microbiome of individuals with HT, marked by a notable reduction in bacteria, such as *Bifidobacterium*, and a concurrent increase in potentially harmful bacteria, such as *Bacteroides*. This alteration in microbiome composition could be pivotal in the development and progression of HT, as it suggests a disruption in critical metabolic pathways (Cayres et al., 2021). Further studies have corroborated these initial findings, observing distinct changes in the gut microbiome of HT patients. For instance, a study has reported a significant reduction in *Bacillaceae* and *Megamonas*, alongside an increase in *Proteobacteria* and *Actinobacteria*. These alterations in the gut microbiome composition are indicative of a decrease in advantageous bacterial species and a relative surge in those that may be detrimental to health, which could play a critical role in the pathogenesis of HT (Zhao et al., 2022). The importance of the “ABC transporter” metabolic pathway has also been highlighted in these studies, as it is found to be highly associated with HT. This suggests a novel avenue that might be targeted in the development of therapeutic strategies for this autoimmune condition.

**Table 1:** Reports that indicate Gut microbiota changes in HT patients from recent 5-year experimental studies in different areas.

Study	Year	Case/control	Increase	Decrease	Patient's Location
Doaa A. Header,etc	2020	20/30	Prevotella, Prevotella/Bacteroides Ratio	A. mucinophilia, Bifidobacterium, Lactobacillus, F. prausnitzii,	Egypt
Francisco J. Tinahones,etc	2020	9/11	*Victivallaceae*	*Faecalibacterium*	Spain
Gislane Lelis Vilela de Oliveira,etc	2021	40/53	Bacteroides	Bifidobacterium	Brazil
JINGYUAN WANG,etc	2022	27/16	Ruminococcus_2, Erysipelotrichia, Cyanobacteria	Bacillaceae, Megamonas	Daqing,China
Qing Lin,etc	2022	42/47	Akkermansia, Lachnospiraceae, Bifdobacterium, Shuttleia, Clostriworthdia	Lachnoclostridium, Bilophila, Klebsiella	Fujian,China
Zhongyan Shan,etc	2024	23/25	Actinobacteriota (p_ Actino bacteriota) , family Prevotellaceae (f_ Prevotellaceae)	Clostridia_UCG-014, Acidaminococcales, Oscillospirales, Desulfovibrionales	Shenyang,China

Continuing this line of inquiry, another study on the gut microbiota diversity in HT patients has revealed significant alterations. There is a consistent decrease such as *Bifidobacterium* and an observed increase including *Klebsiella* (Liu et al., 2022). It also indicated that the development of HT may be promoted by Romboutsia and Haemophilus, which are regulated by free triiodothyronine (T3), while Faecalibacterium and Lachnospiraceae, regulated by free thyroxine (T4), may offer protective effects to the host (Gong et al., 2024). In addition, a study conducted in Egypt has identified a similar pattern in the gut microbiome of patients with autoimmune thyroid diseases (ATD), particularly HT. There is a marked reduction in *Bifidobacterium* and an observed increase in *Bacteroidetes*. This research consistently reports a shift in the gut microbiome composition, indicating a decrease in microbes that are crucial for gut health and immune function, and a rise in bacteria that may contribute to inflammation and disease progression (El-Zawawy et al., 2021). Finally, a Mendelian randomization study has identified a causal link between the gut microbiota and autoimmune thyroiditis (AIT), revealing a significant decrease in bacteria such as *Alcaligenaceae* and *Pasteurellaceae*, alongside an increase in bacteria like *Peptococcaceae* and *Victivallis* (Xiong et al., 2024). According to the IVW method, six gut microbiota species were significantly associated with AIT in the study. Among these, the family Alcaligenaceae, family Pasteurellaceae, family Peptococcaceae, genus Lachnospira, genus Victivallis, and order Pasteurellales demonstrated negative associations with AIT.

The common alterations observed across studies include a significant reduction of bacteria such as *Bifidobacterium, Bacillaceae, Megamonas, Actinobacteriota, and Clostridium*. Concurrently, there is a notable increase of bacteria, including *Bacteroides, Proteobacteria, Actinobacteria, Desulfobacterota, Klebsiella, Peptococcaceae, and Victivallis.* These shifts in the gut microbiome suggest a potential disruption in metabolic pathways and immune balance that may contribute to the pathogenesis of HT, highlighting the possible significance of gut microbiota in the disease’s development and progression (Sadeghpour Heravi, 2024).

The potential decrease in SCFAs may influence T-regulatory cells (Tregs) and could contribute to a Th1/Th2 imbalance, which might lead to an increased production of pro-inflammatory cytokines such as IFN-γ and TNF-α (Wang et al., 2024). These cytokines have been associated with thyroid inflammation and autoimmunity (Ferrari et al., 2023), suggesting a possible link between gut microbiome alterations and thyroid autoimmune conditions (Han et al., 2024). Furthermore, the gut microbiota influences the metabolism of trace elements like selenium, which is crucial for thyroid function and antioxidant defense, and its deficiency may exacerbate HT symptoms (Drutel et al., 2013). Additionally, gut dysbiosis can lead to increased intestinal permeability, allowing for the translocation of bacterial components, such as LPS, which can activate the immune system and trigger the release of additional inflammatory mediators (Brown et al., 2019). This heightened immune response may contribute to the breakdown of immune tolerance and the generation of autoantibodies against thyroid peroxidase and thyroglobulin, which are hallmarks of HT (Tomer and Davies, 1993).

#### 3.1.2 Gut microbiome and Graves’ disease

Graves’ Disease is marked by the presence of thyroid-stimulating hormone receptor (TSHR) antibodies, which are capable of stimulating the TSHR, leading to overproduction of thyroid hormones and hyperthyroidism. These antibodies, known as thyroid-stimulating antibodies (TSAb), are the pathogenic antibodies of GD and are responsible for the clinical manifestations of hyperthyroidism (Struja et al., 2019). The most notable features are weight loss, heat intolerance, and palpitations, which are directly related to the over activity of the thyroid gland such as hyperthyroidism (Song et al., 2023). The global prevalence of Graves’ disease is estimated to be around 1-2%, with recent studies suggesting higher incidence rates among females and certain demographic groups (Troshina et al., 2020). The etiology of Graves’ disease is complex, involving a confluence of genetic susceptibility, environmental factors, and immunological disturbances. In the past five years, research has advanced our understanding of the disease’s triggers. Genetic factors, particularly certain HLA genotypes, have been identified as significant risk factors (Maciel et al., 2001). The role of environmental agents such as excessive iodine intake and exposure to stress has also been increasingly recognized (Xie et al., 2023). Furthermore, the gut microbiota’s composition and its metabolic byproducts are gaining attention for their potential influence on the immune system and the development of GD (Li et al., 2024).

Recent studies have consistently reported a shift in the gut microbiome composition among individuals with GD, with varying reports on the specific types of bacteria affected (Chang et al., 2021). One study has concluded that there is a consistent trend in the gut microbiome of individuals with GD, characterized by a significant decline of bacteria such as *Bacteroidetes* and a rise in bacteria from the Firmicutes group. However, contrasting findings suggest a decrease in Firmicutes and an increase in Bacteroidetes, indicating the complexity of the microbiome’s role in GD. The presence of specific microbiota, such as *Lactobacillus* and *Bacteroides*, has been implicated in thyroid autoimmunity, potentially influencing the immune response against the thyroid gland (Biscarini et al., 2023).

Further research has identified a consistent shift in the gut microbiome composition in GD patients, with a significant decrease in Firmicutes and a relative increase in Proteobacteria and Actinobacteria. The study suggests that Bacillus, Blautia, and Ornithinimicrobium could serve as biomarkers to differentiate GD patients from healthy individuals (Zhao et al., 2022). Additionally, a decrease such as Faecalibacterium and an increase in pro-inflammatory bacteria including Fusobacterium, Sutterella, and Prevotella have been associated with immune system dysregulation in GD (Jiang et al., 2021), contributing to the loss of tolerance to self-antigens and the disease’s pathogenesis (Cornejo-Pareja et al., 2020). The role of SCFA-producing bacteria, including *Bacteroides fragilis*, has been highlighted in another study, with a decrease in these bacteria and a relative increase in bacteria linked to a reduction in SCFAs, particularly propionic acid. This decrease is suggested to contribute to the imbalance between Tregs and T helper 17 (Th17) cells, which is crucial for maintaining immune balance (Su et al., 2020a). In another study, a marked reduction in bacteria such as *Roseburia* and an increase in potentially harmful bacteria including *Bifidobacterium* and *Collinsella* have been observed (Yang et al., 2022a). The research indicates that the abundance of bacteria known for their SCFA production has decreased in GD patients, while there is a relative increase in virulent bacteria. This shift in microbial composition could be pivotal in the immune dysregulation observed in GD (Yang et al., 2022b). An interesting study has pointed out a decrease in bacterial stability and an increase in eukaryotic microbes, particularly fungi and protists, suggesting that physiological changes associated with GD may promote changes in the gut ecosystem. This indicates that eukaryotic microbes could play a more significant role in gut functions than previously thought, hinting at their potential influence on therapeutic interventions for GD (Geng et al., 2024).

Hence, common findings across studies include a significant decrease in bacteria from the Firmicutes group, such as Faecalibacterium and Roseburia, which are known to produce SCFAs and contribute to anti-inflammatory effects. Additionally, there is a relative increase in bacteria from the Bacteroidetes phylum, as well as in bacteria including Enterobacter hormaechei, Chryseobacterium indologenes, and Bifidobacterium species as shown in **Table 2**. These changes are associated with immune system dysregulation and may contribute to the loss of tolerance to self-antigens, playing a role in the pathogenesis of GD.

**Table 2:** Reports that indicate Gut microbiota changes in GD patients from recent 5-year experimental studies in different areas.

Study	Year	Case/control	Increase	Decrease	Patient's Location
Qunye Zhang,etc	2020	58/62	*Yersinia enterocolitica* Bacteroidetes Bacteroides Prevotella Alispes	Firmicutes/Bacteroidetes ratio, Firmicutes	Shandong,China
Francisco J. Tinahones,etc	2020	9/11	Fusobacteriaceae, Fusobacterium and Sutterella	Rikenellaceae	Málaga, Spain
Jiachao Zhang,etc	2021	100/62	Eggerthella lenta Streptococcus parasanguinis, Veillonella parvula, Fuso bacterium mortiferum , Streptococcus salivarius	Faecalibacterium prausnitzii, Butyricimonas faecalis, Bifidobacterium adolescentis , Akkermansia muciniphila	Haikou,China
Dan Li,etc	2021	45/59	Bacteroidetes Bacteroides and Lactobacillus	Firmicutes Blautia [Eubacterium]_hallii_group, Anaerostipes, Collinsella, Dorea, unclassified_f_Peptostreptococcaceae, and [Ruminococcus]_torques_group	Shanghai,China
P Gu,etc	2021	15/14	Lactobacillus, Veillonella and Streptococcus	Synergistetes Phascolarctobacterium Coprococcus Anaerostipes Turicibacter Cloacibacillus Mogibacterium	JiLin,China
J-K Yang,etc	2021	30/32	unidentified -Lachnospiraceae Blautia unidentified_Prevotellaceae	Bacteroides	BeiJing,China
Jang-Jih Lu,etc	2021	55/48	Bacteroidetes Actinomyces_odontolyticus Collinsella Parabacteroides Prevotella_9	Firmicutes Lachnospiraceae	Taiwan,China
JINGYUAN WANG,etc	2022	27/16	Proteobacteria and Erysipelotrichia Cyano Bacteria Prevotella_9, Ruminococ cus_2, and Lachnospiraceae_NK4A136_group	abnormal cocci and Cyanobacteria Megamonas	Harbin,China
Heyuan Ding,etc	2022	191/30	Actinobacteria Bifidobacterium, Collinsella, Pediococcus, N09, 02d06, Enterobacter, Bulleidia, Weissella, Gemella, and Granulicatella	Firmicutes Roseburia, Dialister, Thermus, Slackia (Prevotella), Oxalobacter, Mycoplana, Anaerostipes, Butyricimonas, Corynebacterium and Odoribacter	Shanghai;Zhunyi,China
Miao Zeng,etc	2022	28/11	Bifidobacterium Collinsella Granulicatella	Dialister Roseburia Odoribacter	Zhunyi,China
Marian Ludgate,etc	2023	105/41	Actinobacteria Firmicutes to Bacteroidetes (F:B) ratio	Bacteroidetes	4 European countries
Jixiong Xu,etc	2023	65/33	Bacilli at the class level; Lactobacillales at the order level; Streptococcaceae at the family level; and Streptococcus, Veillonella, and Erysipelatoclostridium at the genus level	Peptostreptococcaceae, Christensenellaceae, Marinifilaceae, and Rikenellaceae at the family level and the abundance of 10 genera, such as Roseburia, Romboutsia, Lachnospira, and Eubacterium ventriosum, at the genus level	Nanchang,China
Dong-Jun Lim,etc	2024	29/230	Bacteroidota	Firmicutes, Roseburia, Lachnospiraceaea, Sutterella, Escherichia-shigella, Parasuterella, Akkermansia, and Phascolarctobacterium Firmicutes/Bacteroidetes ratio	Seoul,Republic of Korea
Huijuan Yuan,etc	2024	39/20	Lachnospiraceae and Eggerthellaceae, Piptocephalidaceae and Spizellomycetaceae	Saccharomycetales incertae sedis,	Henan,China

### 3.2 The microbiota’s broader impact on non-autoimmune thyroid diseases

Non-autoimmune thyroid diseases, which mainly include thyroid nodules, thyroid cancer, have distinct etiologies and prevalence rates that vary by age, sex, and geographic location. Defined as abnormal growths within the thyroid gland, thyroid nodules are increasingly detected due to advances in imaging technology, with a reported prevalence of 19–55% in the general population, rising to 50% in individuals over 50 years (Tran et al., 2023). And the incidence of thyroid cancer has been rising, with a global age-standardized rate of 1.6 per 100,000 population, making it the fastest-growing cancer in the United States (Pan et al., 2020). The rise is attributed to improved detection and diagnostic techniques. Meanwhile, excessive iodine intake can cause or exacerbate thyroid disorders. The prevalence of iodine-induced hyperthyroidism is particularly high in regions with adequate iodine intake, affecting approximately 0.5–1% of the population (Zimmermann and Andersson, 2012). The relationship between the gut microbiome and non-autoimmune thyroid diseases is an emerging area of research. While the majority of studies have focused on autoimmune thyroid diseases, recent work has begun to explore the potential links between the gut microbiome and non-autoimmune conditions.

#### 3.2.1 Gut microbiome and thyroid nodules

Thyroid nodules are defined as discrete lesions within the thyroid gland that are radiologically distinct from the surrounding thyroid tissue (Durante et al., 2023). They can be solitary or multiple, and may be either cystic or solid in composition. The development of thyroid nodules is multifactorial, involving genetic predisposition, environmental exposures, and hormonal factors. Iodine deficiency, a classic environmental risk factor, has been extensively studied and is known to contribute to nodule formation (Su et al., 2020b). With the advent of high-resolution ultrasound, the detection rate of thyroid nodules has increased significantly. It is estimated that over 60% of the general population has thyroid nodules, many of which are asymptomatic (Grani et al., 2022). Only a small fraction of thyroid nodules are malignant. The risk of thyroid cancer within a nodule is influenced by factors such as nodule size, growth rate, and the presence of suspicious ultrasound features (Yoon et al., 2008). There is growing interest in the role of inflammation and the gut microbiome in thyroid nodule development. Chronic inflammation may increase the risk of developing nodules, and there is preliminary evidence suggesting that an altered gut microbiome could be implicated (Wu et al., 2023).

In patients with thyroid nodules (TN), the gut microbiome exhibits a reduction in microbes such as Butyrivibrio, Coprococcus comes, Coprococcus catus, Roseburia hominis, Eubacterium eligens, and Faecalibacterium prausnitzii (Li et al., 2021). Additionally, metabolic pathways related to butyrate production are decreased, while those associated with amino acid biosynthesis are increased, suggesting a dysregulation in gut microbial metabolism that may influence the thyroid function as shown in **Table 3**. In another study focusing on thyroid nodules, the gut microbiome is characterized by a significant increase in the relative abundance of *Neisseria* and *Streptococcus* (Zhang et al., 2019), and a notable decrease in microbes like *Butyricimonas* and *Lactobacillus*. The study also observed alterations in metabolic pathways related to lipid digestion and steroid biosynthesis, which may be integral to the pathogenesis of thyroid nodules. These findings suggest a complex interplay between gut microbiota imbalance and thyroid nodule development. Another study identifies an increased abundance of bacteria such as Class Mollicutes and Phylum Tenericutes in TN patients, while observing a decrease in the Genus Eubacterium fissicatena group and Genus *Lachnospiraceae UCG008* (Hu et al., 2024). In addition, in TN, a common shift is observed with a consistent decrease in Butyrivibrio, Coprococcus, Eubacterium, and Faecalibacterium prausnitzii, and an increase in bacteria, particularly Neisseria and Streptococcus. Additionally, there is a reduction in metabolic pathways for butyrate production and an upregulation in amino acid biosynthesis pathways, indicating a disrupted gut microbial metabolism that may affect thyroid function and nodule development. Moreover, the presence of *Helicobacter pylori*, while classically associated with gastric issues, has also been implicated in thyroid nodule formation, possibly due to its systemic inflammatory effects (Shen et al., 2013).

**Table 3:** Reports that indicate Gut microbiota changes in TN patients from recent 5-year experimental studies in different areas.

Study	Year	Case/control	Increase	Decrease	Patient's Location
LeiZhang,etc	2019	18/36	Neisseria , Streptococcus	Butyricimonas, Lactobacillus	Shandong,China
Suying Ding,etc	2021	196/283	Coprococcus comes, Coprococcus catus, Roseburia hominis, Eubacterium eligens	Bacteroides ovatus , Eggerthella unclassified	Zhengzhou,China
Ning Ma,etc	2022	9/15	genre uncultured Candidatus Saccharibacteria bacterium, unclassified Clostridiales bacterium feline oral taxon 148, Treponema, unclassified Prevotellaceae, Mobiluncus, and Acholeplasma	Rhodobacteraceae and Aggregatibacter	Tianjin,China

A balanced immune response is essential for preventing autoimmune reactions that could potentially lead to the development of thyroid nodules. The metabolic activities of gut microbiota directly affect thyroid hormone levels. SCFAs produced by gut bacteria can influence the conversion of T4 to T3, the active form of thyroid hormone. This metabolic interplay is vital for regulating thyroid function, and any disruption can lead to hormonal imbalances that may contribute to the formation of thyroid nodules (Furusawa et al., 2013). Bile acids, essential for the digestion and absorption of fats, are also metabolized by the gut microbiome. Alterations in bile acid metabolism due to changes in gut microbiota composition can impact the enterohepatic circulation of thyroid hormones, thereby influencing thyroid function and potentially contributing to the development of thyroid nodules (Berinde et al., 2024).

An imbalance in the gut microbiome can disrupt the tight junctions of the intestinal epithelium, leading to increased gut permeability (Di Vincenzo et al., 2024). This “leaky gut” allows for the translocation of bacterial components, such as LPS, and other antigens into the systemic circulation. These translocated antigens can act as triggers for the immune system. Once in the systemic circulation, these bacterial components can activate immune cells, such as macrophages and dendritic cells, leading to the release of pro-inflammatory cytokines like TNF-α and IL-6 (Liu et al., 2021). This activation can initiate a cascade of inflammatory responses that, if persistent, result in chronic inflammation (Zhang et al., 2023). Chronic inflammation can cause cellular stress in the thyroid gland, leading to the activation of stress pathways and the production of reactive oxygen species (ROS) (Macvanin et al., 2022). This stress can induce DNA damage and alterations in thyroid follicular cells, potentially contributing to the development of thyroid nodules (Jiao et al., 2022). The inflammation and immune activation associated with a leaky gut can also affect the production and regulation of thyroid hormones (Zhu et al., 2024). This can disrupt the normal feedback loop of the HPT axis, leading to hormonal imbalances that may further contribute to nodule formation (Bargiel et al., 2021). Some studies suggest that certain bacterial components, such as LPS, may have a direct effect on thyroid cells, influencing their growth and function (Xu et al., 2013). This could potentially lead to the formation of thyroid nodules through mechanisms that are still under investigation (Ludgate et al., 2024).

The gut microbiome’s influence on thyroid function extends beyond cancer, affecting the development of thyroid nodules through neural, hormonal, and immune pathways (Knezevic et al., 2020). It can modulate the production of cytokines like IL-1β, IL-6, and TNF-α, which are known to influence thyroid cell proliferation (Takahashi et al., 2020). An overproduction of these pro-inflammatory cytokines can lead to excessive cell growth, potentially contributing to nodule formation. Additionally, chemokines produced in response to the gut microbiome, such as CCL2 and CXCL8 (Turan and Turksoy, 2021), can recruit immune cells like macrophages and neutrophils to the thyroid gland, where they may contribute to inflammation and nodule formation through the release of ROS and other mediators. The gut microbiome also impacts thyroid hormone metabolism by supporting selenium-dependent processes, and its metabolic activity can convert dietary components (Jiang et al., 2022), including goitrogens, into metabolites that affect thyroid function and potentially lead to nodule formation (Vatseba et al., 2002).

#### 3.2.2 Gut microbiome and thyroid cancer

Thyroid cancer is a malignant neoplasm originating from the follicular or parafollicular cells of the thyroid gland (Cabanillas et al., 2016). It is the most common endocrine malignancy and is classified into several types, including papillary, follicular, medullary, and anaplastic carcinoma, with papillary thyroid cancer being the most prevalent (Haugen et al., 2016). The reasons behind this increase are multifactorial and include environmental factors, changes in diagnostic practices, and lifestyle behaviors, Iodine intake, radiation exposure, and genetic predisposition are well-established risk factors (Saenko and Mitsutake, 2024). The global age-standardized incidence rate of thyroid cancer is approximately 5.6 per 100,000, with higher rates observed in females and individuals aged 45-54 years (Alagoz et al., 2024). The disease is more common in developed countries, although there is a growing trend in developing regions as well.

The role of the gut microbiome in thyroid cancer development is an emerging area of research, with some studies suggesting that alterations in the gut microbiota may contribute to cancer progression (Hou et al., 2023), as shown in **Table 4**. Furthermore, the metabolic pathways influenced include ketogluconate metabolism and the pentose phosphate pathway, indicating a complex interplay between gut microbiota and thyroid cancer progression (Hou et al., 2023). Studies have characterized by a marked reduction in bacteria such as *g_Christensenellaceae_R-7_group and g_Eubacterium_coprostanoligenes_group*, which are crucial for lipid metabolism homeostasis. Conversely, there is a relative increase in bacteria associated with the Bacteroides enterotype, which may be linked to a high-fat diet and potentially contribute to the development of TC. The research also indicates that metabolic pathways related to lipid digestion and steroid biosynthesis are dysregulated in TC patients, suggesting that gut microbiota imbalance and lipid metabolic disorders could be integral to the pathogenesis of thyroid cancer (Lu et al., 2022).

**Table 4:** Reports that indicate Gut microbiota changes in thyroid cancer patients from recent 5-year experimental studies in different areas.

Study	Year	Case/control	Increase	Decrease	Patient's Location
Lei Zhang,etc	2019	20/36	Neisseria (p < 0.001) and Streptococcus (p < 0.001)	Butyricimonas, Lactobacillus	Qingdao,China
Xiao-Ran Li,etc	2021	25/0	Pseudomonas mucidolens and Escherichia fergusonii	-	Kunming,China
Dan Li,etc	2022	50/58	Megamonas Proteobacteria	g_Christensenellaceae_R-7_group, g_Eubacterium_coprostanoligenes_group Prevotella_9	Shanghai,China
Dan Li,etc	2022	90/90	g:Fusobacterium and g:Alistipes	Prevotella g:Hungatella and g:Phascolarctobacterium.	Shanghai,China
Ning Ma,etc	2022	14/15	Alloprevotella, Anaeroglobus, Acinetobacter, unclassified Bacteroidales, and unclassified Cyanobacteriales	Haemophilus, Lautropia, Allorhizobium Neorhizobium Pararhizobium Rhizobium, Escherichia Shigella, and unclassified Rhodobacteraceae	JinLin;Qingdao,China
Yunwei Wei,etc	2022	62/40	porphyromonas, fusobacterium, and treponema_2	streptococcus	Guangzhou,China
Weg M. Ongkeko,etc	2023	453/54	Botrytis cinerea	Phialophora verrucosa, Boletinellus merulioides and Bipolaris sorokiniana	San Diego,USA
Yong Meng,etc	2023	211	Ruminiclostridium9 , class Mollicutes , genus RuminococcaceaeUCG004, genus Paraprevotella, and phylum Tenericutes	Actinobacteria	Mibiogen database
Yufang Bi,etc	2023	6699/1 620 354	RuminococcaceaeUCG004 Ruminococcus2 Fusicatenibacter genus Butyrivibrio genus Olsenella genus RuminococcaceaeUCG003 genus Oscillospira genus and Streptococcaceae family	Bacillales order and Proteobacteria phylum	Global Biobank Meta-analysis Initiative
Ting Yu,etc	2023	18,340 / 6,699	Holdemanella Butyrivibrio, Fusicatenibacter, Oscillospira, Ruminococcus2 , and Terrisporobacter	Bacillales	The Global Biobank Meta-Analysis Initiative
Haibo Cheng,etc	2024	18340	Christensenellaceae, Family Victivallaceae, Genus Methanobrevibacter, Genus Ruminococcus2 , Genus Subdoligranulum, Phylum Verrucomicrobia, Class Betaproteobacteria,Genus Sutterella	Genus Ruminococcus2	The MiBioGen consortium's genome-wide association studies (GWAS)
Lijun Yao,etc	2024	649/431	Lachnospiraceae UCG010 Mollicutes/Tenericutes	-	The GLOBOCAN (2020) database

Patients with papillary thyroid carcinoma (PTC) following radioactive iodine (131I) therapy, characterized by a notable decrease in bacteria such as Firmicutes, including the genera *Faecalibacterium*, *Lachnospira*, and *Lachnospiraceae NK4A136* group. Conversely, there is a relative increase such as Bacteroidetes, with higher abundances of the families Prevotellaceae, Veillonellaceae, and the genus Prevotella_9. Additionally, the research identifies a predictive model for response to 131I therapy, highlighting the role of gut microbiota as a potential predictor for therapeutic outcomes in PTC patients (Zheng et al., 2023). The study consistently reports a decrease bacteria such as the *RuminococcaceaeUCG004* genus and Streptococcaceae family, alongside an increase in bacteria like the *Holdemanella* genus, which are suggested to be influenced by the presence of thyroid cancer. A significant decrease in bacteria such as Firmicutes and an increase in bacteria, including *Proteobacteria (* Liu et al., 2021). The microbial metabolism in both gut and thyroid tissues appears to be influenced by the presence of TC, suggesting a complex interplay between the tumor microenvironment and the microbiota. Notably, in another research, there was a decrease such as *Faecalibacterium*, *Ruminococcaceae_UCG-002*, and *Phascolarctobacterium*, which are known to produce SCFAs.

Conversely, there was a relative increase including those from the Proteobacteria phylum, which is often associated with dysbiosis (Yu et al., 2022). Another study uncovers a distinct dysregulation of the intratumor mycobiome and archaeome in PTC, with significant differences observed across subtypes and genders (John et al., 2023). While a general overabundance of fungal species was noted in PTC tissues compared to normals, specific subtypes such as the Tall Cell variant demonstrated a more pronounced dysregulation. The study indicates a potential role of the mycobiome in PTC oncogenesis, with certain fungal species like *Volvariella volvacea* showing an overabundance in BRAF V600E-positive tumors, suggesting a link between microbial dysregulation and oncogenic pathways. However, the exact mechanisms and clinical implications require further *in vitro* studies to confirm these associations.

A study provides evidence of a causal relationship between the gut microbiota and differentiated thyroid carcinoma (DTC), identifying specific shifts in microbial composition. It reports a significant decrease in the phylum Actinobacteria, which is associated with a reduced risk of DTC. In contrast, an increase in bacteria such as the genus *Ruminiclostridium 9*, class Mollicutes, genus *Ruminococcaceae UCG004*, genus *Paraprevotella*, and phylum Tenericutes is linked to a higher risk of DTC (Quan et al., 2023). These findings suggest that alterations in the gut microbiome may play a role in the development of DTC, potentially affecting pathways related to thyroid function and immune response modulation.

Another research reveals significant alterations in the salivary microbiota of patients with thyroid cancer, suggesting a potential connection between oral microbiota dysbiosis and thyroid conditions (Jiao et al., 2022). It identifies a higher alpha-diversity in saliva microbiota for these patients compared to healthy controls, with specific genera such as *Alloprevotella*, *Anaeroglobus*, and *Acinetobacter* being significantly enriched in the thyroid cancer group. Additionally, the study finds certain clinical indicators, including TSH and TPOAb levels, to be correlated with the saliva microbiome, indicating a possible interaction between thyroid diseases and oral microbiota that warrants further investigation. While the other research concludes that differentiated DTC patients undergoing radioiodine therapy exhibit a distinct oral microbiota composition associated with xerostomia, marked by a significant increase in bacteria and a decrease in beneficial species (Lin et al., 2022a). Notably, genera such as *Porphyromonas*, *Fusobacterium*, and *Treponema_2* are enriched, potentially exacerbating inflammation, while bacteria like Streptococcus are diminished. Additionally, the study reveals a shift in metabolic pathways, with those related to inflammation and antioxidant mechanisms being upregulated in the oral microbiome of affected patients, suggesting a pro-inflammatory environment that may contribute to xerostomia severity.

There is a study that employs Mendelian randomization to establish a potential causal link between gut microbiota and thyroid cancer, identifying specific microbial taxa that may influence thyroid cancer risk (Zhu et al., 2023). It finds that certain bacteria, including the genus *Butyrivibrio*, *Fusicatenibacter*, *Oscillospira*, *Ruminococcus2*, and *Terrisporobacter*, are associated with an increased risk of thyroid cancer, while the genus *Olsenella* and *Ruminococcaceae UCG004* are linked to a reduced risk. Additionally, the study suggests that the presence of the genus *Holdemanella* may increase, and the order Bacillales may decrease following the development of thyroid cancer, indicating a possible reverse causal effect.

In the treatment study, a significant alteration in the gut microbiome of a patient with anaplastic thyroid cancer (ATC) treated with pembrolizumab was observed; there was a notable increase in the abundance of bacteria from the orders Bacteroidales, specifically Bacteroidaceae and Rikenellaceae, along with enrichment in Clostridiales order members such as Ruminococcaceae, Veillonellaceae, and Lachnospiraceae (Aghajani et al., 2019). Additionally, the patient’s gut microbiome exhibited a significant increase in alpha diversity following the initiation of checkpoint therapy, suggesting a potential role of the microbiome in modulating the response to immunotherapy. The study implies that the gut microbiota may have a substantial influence on the efficacy of cancer treatments, particularly immunotherapies targeting the PD-1 pathway. Thyroid cancer is associated with a common shift in the gut microbiome, where bacteria such as Firmicutes, particularly Faecalibacterium, Lachnospira, and Ruminococcaceae, are significantly decreased. Conversely, there is a relative increase in Bacteroidetes and Proteobacteria, which may contribute to cancer progression and affect the efficacy of treatments like radioactive iodine therapy.

Metabolic landscape in thyroid cancer patients is significantly influenced by the gut microbiota. SCFAs such as butyrate, acetate, and propionate, which are produced by bacteria like Faecalibacterium prausnitzii, have been shown to have anti-inflammatory and antitumor effects (Zhang et al., 2019). However, in thyroid cancer, a decrease in SCFA-producing bacteria has been noted, which may lead to a reduction in these protective metabolites. Additionally, bile acid metabolism mediated by the gut microbiota can influence cancer cell growth, with secondary bile acids like deoxycholic acid (DCA) promoting tumorigenesis (Yano et al., 2008). These metabolic changes can disrupt thyroid hormone homeostasis and contribute to the pathogenesis of thyroid cancer.

The gut microbiota plays a pivotal role in modulating the host’s immune system, which has significant implications for thyroid cancer. It can influence the balance between pro-inflammatory and anti-inflammatory cytokines, crucial for cancer immunosurveillance. For instance, certain gut bacteria can produce IL-10, promoting a Treg response that may suppress antitumor immunity (Arpaia et al., 2013). On the other hand, an increase in pro-inflammatory bacteria can lead to the production of cytokines such as IL-17 and IL-23, enhancing the immune response against cancer cells. This bidirectional communication between the gut microbiota and the immune system is intricate and can either support or hinder cancer treatment efficacy (Riquelme et al., 2019).

**Figure 2:**
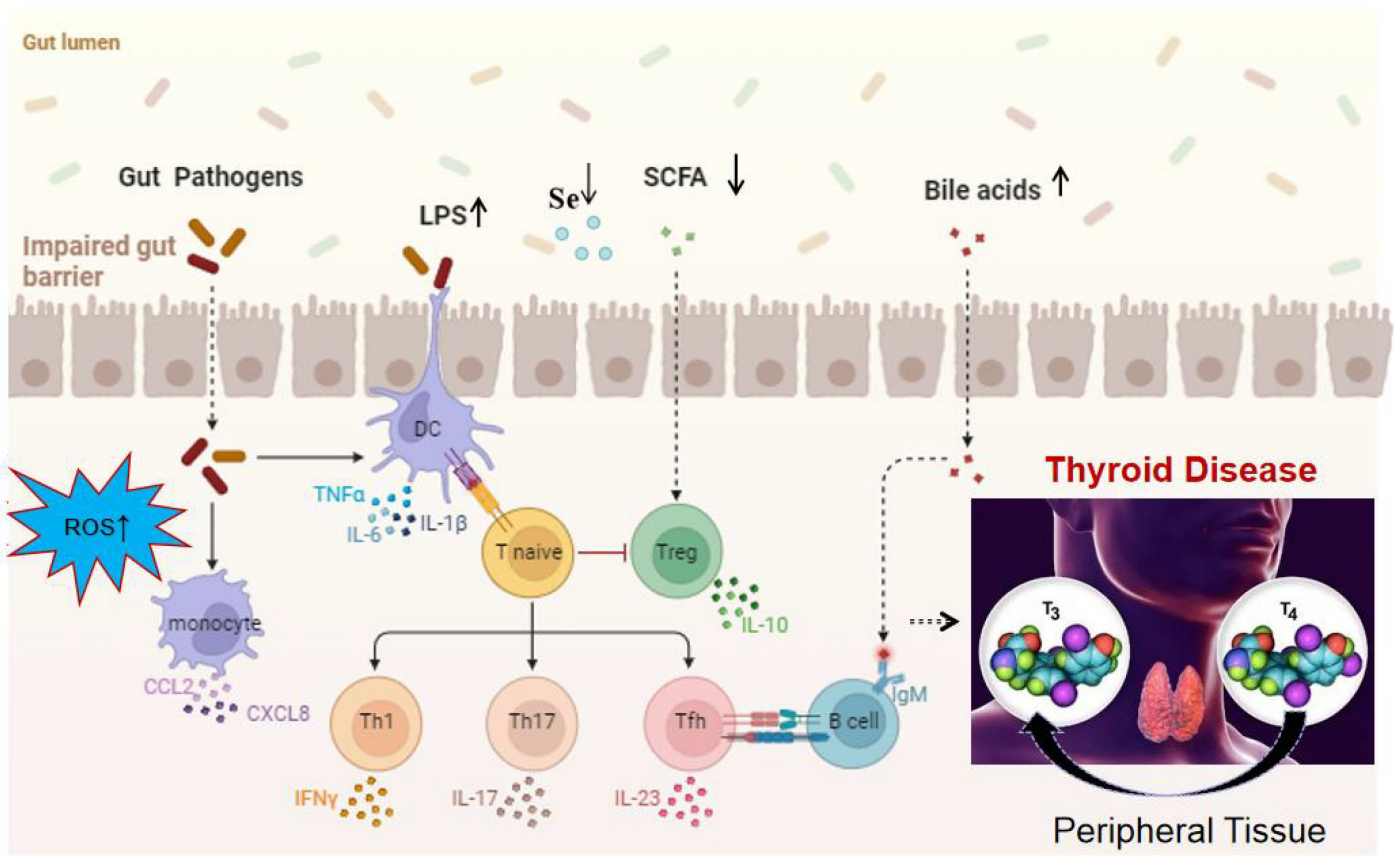
Mechanistic overview of the impact of gut microbiota on thyroid function. It highlights the role of ROS, LPS, SCFAs, bile acid metabolism, cytokine production, and chemokine-induced immune cell recruitment, increased intestinal permeability, changes in selenium levels in disrupting the gut microbiome, ultimately leading to thyroid disorders. These factors contribute to a cascade of metabolic and immunological effects that can alter thyroid function and promote the pathogenesis of thyroid diseases.

In summary, the disruption of the gut microbiome is implicated in thyroid-related diseases through several key mechanisms. Initially, alterations in the microbial composition result in a reduction of SCFAs, impacting T cell activity and the secretion of inflammatory cytokines such as IFN-γ, TNF-α, chemokine ligands (CCL-2 and CXCL-8), IL-17, IL-23, and IL-10. This in turn affects the conversion of T4 to the more active form, T3. Additionally, the gut microbiome influences the metabolism of bile acids, which may contribute to the development of thyroid nodules and cancer. Furthermore, variations in the gut microbiota can affect selenium absorption, a critical component for the deiodinase enzymes that convert T4 to T3, potentially leading to HT. The compromised intestinal permeability, often seen in an imbalanced gut microbiome, facilitates the translocation of bacterial components like LPS, triggering an immune response that can result in increased levels of autoantibodies and the associated inflammation characteristic of thyroid disorders. Lastly, the microbiome’s influence on the production of ROS within cells may further promote the pathogenesis of thyroid diseases, as illustrated in the **Figure 2**.

### 3.3 Clinical frontiers: probiotic adjuvant therapeutic effect

#### 3.3.1 Hashimoto’s thyroiditis

A recent study suggests a comprehensive approach to assessing the impact of nutritional intervention with the probiotic *Lactiplantibacillus plantarum* 299v on the nutritional status and quality of life of patients with Hashimoto’s Thyroiditis (Osowiecka et al., 2023). The hypotheses are that personalized nutrition education may improve nutritional status and quality of life, and that the Lp299v strain may enhance these effects, particularly on quality of life and blood anti-TPO parameters. While the research is on the going and provides a detailed study design and methodology, it does not include results or conclusions from the research itself. Therefore, based on the research alone, we cannot determine whether the probiotic is effective in treating Hashimoto’s Thyroiditis. And in a subsequent meta-analysis encompassing two randomized controlled trials that explored the impact of probiotic, prebiotic, and symbiotic treatments in individuals with hypothyroidism, there was a slight reduction in TSH levels, though this was not statistically significant, along with a statistically significant alteration in fT4 levels. The findings hint that the regular use of these biological therapies might offer only minimal advantages to patients suffering from primary hypothyroidism (Zawadzka et al., 2023). Synbiotic therapy for hypothyroid patients may enhance thyroid function, indicating a positive role for gut microbiome modulation in treatment. However, more extensive and long-term studies are essential to validate these preliminary benefits and determine optimal symbiotic formulations for clinical use (Lin et al., 2022b).

Meanwhile, there is a IMITHOT trial represents a pioneering initiative, embarking on a double-blinded, randomized controlled journey to evaluate the efficacy of fecal microbiota transplantation on bolstering thyroid function in subjects with subclinical autoimmune hypothyroidism in the Netherlands (Fenneman et al., 2023). This innovative study marks the inaugural exploration into harnessing the restorative capabilities of FMT to potentially arrest the advancement of the HT disease and mitigate autoimmune responses. Another research showed that FMT from HT patients intensified thyroiditis and immune dysregulation in CBA/J mice, as evidenced by elevated serum TgAb levels and disrupted T-cell balance. Conversely, the introduction of microbiota from healthy donors, along with hydrogen sulfide (H2S), mitigated these effects, indicating a therapeutic potential of FMT in modulating thyroiditis severity. The study highlights the FMT’s role in influencing the gut-thyroid axis and underscores the importance of the gut microbiome in thyroiditis pathogenesis (Zhang et al., 2024).

#### 3.3.2 Graves’ disease

In a 6-month clinical study, patients with GD were assigned to three treatment groups, including methimazole alone or in combination with black beans and probiotic *Bifidobacterium longum*. The study aimed to assess the impact of these treatments on thyroid function and the intestinal microbiota. Notably, the addition of probiotics to methimazole therapy not only improved clinical thyroid indices but also significantly reduced TRAb levels to those found in healthy individuals, suggesting a potential therapeutic role for probiotics in managing GD by modulating the gut microbiota and influencing neurotransmitters and trace elements via the gut-brain and gut-thyroid axes (Shu et al., 2024). A ten-week symbiotic intervention demonstrated no significant impact on depression and TSH levels in hypothyroid patients but positively influenced blood pressure and overall quality of life (Han et al., 2021). Vancomycin can alleviate Graves’ Disease symptoms, but FMT from patients with Graves’ Disease may worsen the condition in mouse models. Probiotics, such as Bifidobacterium longum, are being explored as a means to modulate the gut microbiota and ameliorate disease symptoms (Hou et al., 2021). A clinical trial found that *VSL#3^®^* probiotic supplementation alongside LT4 did not directly affect thyroid functional compensation in primary hypothyroidism patients (Spaggiari et al., 2017). The study suggests a potential probiotics-mediated influence that may stabilize serum hormonal levels, indicating a possible role for gut microbiome modulation in thyroid hormone homeostasis.

The therapeutic potential of FMT in GD has demonstrated encouraging outcomes, as evidenced by recent studies (Su et al., 2020b; Moshkelgosha et al., 2021; Khakisahneh et al., 2022). The FMT demonstrated that mice given microbiota from individuals with primary hypothyroidism exhibited a reduction in total thyroxine levels (Su et al., 2020b). In an animal model, the transplantation of microbiota from animal donors led to significant changes in the gut microbiome following FMT. This was accompanied by a notable reduction in serum T3 and T4 levels, an upregulation of hepatic type 2 deiodinase expression, and an improved restoration of the metabolic rate disturbed by hypothyroidism back to its normal state (Khakisahneh et al., 2022). Additionally, FMT involving the transfer from human donors to animal recipients induced shifts in the gut microbiota, including a decrease in Bacteroides abundance and an enhancement of microbial richness indices. These findings suggest that FMT may serve as a corrective measure for microbiome imbalances associated with the disease (Hou et al., 2021). In the context of immune dysregulation, GD is marked by an elevated presence of Th17 cells and a diminished population of Tregs, indicating a disrupted immune homeostasis (Li et al., 2016). Furthermore, the introduction of prebiotics like berberine and probiotics such as *Bifidobacterium longum* alongside traditional treatments like methimazole has shown promise in modulating the gut microbiome and improving therapeutic outcomes for GD patients (Han et al., 2021). Berberine, acting as a potential prebiotic, has been shown to modulate the gut microbiota by increasing the abundance of beneficial bacteria such as *Lactococcus lactis* and decreasing the abundance of pathogenic bacteria like *Enterobacter hormaechei* and *Chryseobacterium indologenes*, thereby improving the therapeutic outcomes for GD patients when combined with methimazol (Cheng et al., 2022). The combination of berberine with methimazole has demonstrated the ability to regulate metabolic pathways in the gut microbiota, significantly upregulating the synthesis of enterobactin, which is essential for iron uptake and may contribute to the restoration of normal thyroid function in GD patients (Yang et al., 2023).

#### 3.3.3 Thyroid nodules and thyroid carcinoma

Previous findings suggest that the gut microbiota’s composition is linked to both thyroid cancer and thyroid nodules, potentially enhancing clinical diagnostic approaches and informing the development of probiotic therapies for these conditions (Zhang et al., 2019). However, there are few research on the Thyroid nodules with probiotic. In a study involving fifty thyroid carcinoma patients who underwent thyroidectomy, the administration of a probiotic mixture significantly alleviated postoperative symptoms such as fatigue, constipation, weight gain, and dry mouth, and improved dyslipidemia by modulating fecal and serum LPS levels and plasma lipid profiles. Probiotic treatment also restored microbial diversity in the gut and oral cavity, with specific shifts in microbial composition linked to the alleviation of these postoperative complications (Lin et al., 2022b). Utilizing probiotics may promote a better gut microbiota, potentially exerting an indirect effect on thyroid cancer (Fröhlich and Wahl, 2019).

Thyroid cancer is frequently characterized by a decrease in Lactobacillus and Bifidobacteria populations. In a murine model, the addition of L. reuteri led to an increase in T4 levels, a response mediated by the activation of interleukin-10 and the bolstering of regulatory T cells (Zhao et al., 2018). The administration of Lactobacillus-based probiotics in broiler chickens resulted in an enhancement of both T3 and T4 levels (Sohail et al., 2010). Probiotics have been shown to stabilize serum hormone levels and modulate the deconjugation of iodothyronines via specific bacterial enzymes, including sulfatases and β-glucuronidases (Knezevic et al., 2020). Probiotics are capable of accumulating essential trace elements like zinc, selenium, and copper, and incorporating them into vital organic compounds. The modulation of the oral and gut microbiota by probiotics may lead to a reduced incidence of complications in thyroid cancer patients. A probiotic supplement comprising *B. infantis*, *L. acidophilus*, *E. faecalis*, and *B. cereus* has been found to alleviate complications in thyroid cancer and to rejuvenate the oral and gut microbiota (Lin et al., 2022b). The use of probiotics has been associated with a reduction in oral *Prevotella_9*, *Fusobacterium*, *Haemophilus*, and *Lautropia*, and an increase in gut *Holdemanella*, *Coprococcus_2*, and *Enterococcus*. Probiotic intake facilitates the regulation of the gut microbiota and associated metabolites, which engage with neurotransmitters via the gut-thyroid axis, thereby enhancing thyroid function (Leenhardt et al., 2019). The supplementation of prebiotics brings about targeted modifications in the gastrointestinal microbiota, reshaping microbial ecology and fermentation dynamics (Rehman et al., 2008). The addition of mannooligosaccharide prebiotics has been shown to stimulate the proliferation of *Lactobacillus* and *Bifidobacterium*, bacteria that are integral to thyroid function (Baurhoo et al., 2007). Introducing prebiotics into the diet represents an alternative strategy for enhancing gut microbiota composition, potentially leading to improved thyroid function and a reduced risk of thyroid cancer (Jia et al., 2021). The utilization of probiotic bacteria fails to counteract the detrimental effects of radioactive iodine-131 therapy on Thyroid carcinoma.

## 4 Discussion

This comprehensive review illuminates the complex dynamics between the gut microbiome and thyroid diseases, revealing distinct microbial signatures associated with various thyroid pathologies. Through an analysis of the latest research, we’ve observed a resounding trend: a diminution in bacteria genera such as *Bifidobacterium* and an upsurge in potentially detrimental ones like Bacteroides and Proteobacteria. These shifts in microbial composition, as depicted in **Figure 1**, are implicated in the intricate pathogenesis of thyroid diseases, influencing metabolic pathways and immune responses that are vital for thyroid function. **Figure 2** provides a visual synthesis of the mechanisms by which alterations in the gut microbiome can exert effects on thyroid health, underscoring the metabolic and immunological interplay that is disrupted in disease states. The production of SCFAs, critical for maintaining immune balance and thyroid hormone regulation, is notably compromised in these conditions. The review also brings into focus the burgeoning therapeutic horizons offered by probiotics and FMT. The evidence presented points towards a promising role for these interventions in recalibrating the gut microbiota, thereby modulating thyroid function and potentially improving patient outcomes. Probiotic therapies, as highlighted, could serve to replenish bacteria, enhancing the production of SCFAs and supporting the immune-regulatory network.

Looking to the future, the trajectory of research in this domain is poised for significant growth. We anticipate a surge in personalized medicine approaches, with the gut microbiome serving as a pivotal biomarker for diagnostic and therapeutic strategies. The integration of microbiome-based interventions with traditional treatments is foreseen to engender a more efficacious and nuanced management paradigm for thyroid diseases. Additionally, the long-term impact of the gut microbiome during critical developmental stages and the influence of environmental factors such as diet and lifestyle on thyroid health are areas ripe for further exploration. In conclusion, this review encapsulates the burgeoning evidence base for the role of the gut microbiome in thyroid diseases and the potential of probiotic and FMT therapies. It underscores the imperative for continued research to unravel the full therapeutic potential of microbiome modulation, heralding a new era in personalized thyroid disease management.
